# Tumor control and trigeminal dysfunction improvement after stereotactic radiosurgery for trigeminal schwannomas: a systematic review and meta-analysis

**DOI:** 10.1007/s10143-020-01433-w

**Published:** 2020-11-13

**Authors:** Iulia Peciu-Florianu, Jean Régis, Marc Levivier, Michaela Dedeciusova, Nicolas Reyns, Constantin Tuleasca

**Affiliations:** 1 Neurosurgery and Neurooncology Service, Centre Hospitalier Regional Universitaire de Lille, Roger Salengro Hospital, Lille, France; 2 Stereotactic and Functional Neurosurgery Service and Gamma Knife Unit, CHU Timone, Marseille, France; 3 Department of Clinical Neurosciences, Neurosurgery Service and Gamma Knife Center, Lausanne University Hospital (CHUV), Lausanne, Switzerland; 4 Faculty of Biology and Medicine and Centre Hospitalier Universitaire Vaudois (CHUV), Department of Clinical Neurosciences, Neurosurgery Service and Gamma Knife Center, University of Lausanne, Lausanne, Switzerland; 5 First Faculty of Medicine, Charles University in Prague, Prague, Czech Republic; 6 Department of Neurosurgery and Neurooncology, Military University Hospital Prague, Prague, Czech Republic; 7 Signal Processing Laboratory (LTS 5), Ecole Polytechnique Fédérale de Lausanne (EPFL), Lausanne, Switzerland

**Keywords:** Trigeminal schwannoma, Radiosurgery, Gamma Knife, Trigeminal neuralgia

## Abstract

Trigeminal nerve schwannomas (TS) are uncommon intracranial tumors, frequently presenting with debilitating trigeminal and/or oculomotor nerve dysfunction. While surgical resection has been described, its morbidity and mortality rates are non-negligible. Stereotactic radiosurgery (SRS) has emerged with variable results as a valuable alternative. Here, we aimed at reviewing the medical literature on TS treated with SRS so as to investigate rates of tumor control and symptomatic improvement. We reviewed manuscripts published between January 1990 and December 2019 on PubMed. Tumor control and symptomatic improvement rates were evaluated with separate meta-analyses. This meta-analysis included 18 studies comprising a total of 564 patients. Among them, only one reported the outcomes of linear accelerators (Linac), while the others of GK. Tumor control rates after SRS were 92.3% (range 90.1–94.5; *p* < 0.001), and tumor decrease rates were 62.7% (range 54.3–71, *p* < 0.001). Tumor progression rates were 9.4% (range 6.8–11.9, *p* < 0.001). Clinical improvement rates of trigeminal neuralgia were 63.5% (52.9–74.1, *p* < 0.001) and of oculomotor nerves were 48.2% (range 36–60.5, *p* < 0.001). Clinical worsening rate was 10.7% (range 7.6–13.8, *p* < 0.001). Stereotactic radiosurgery for TS is associated with high tumor control rates and favorable clinical outcomes, especially for trigeminal neuralgia and oculomotor nerves. However, patients should be correctly advised about the risk of tumor progression and potential clinical worsening. Future clinical studies should focus on standard reporting of clinical outcomes.

## Introduction

Trigeminal nerve schwannomas (TS) are rare, representing less than 1% of all intracranial tumors [39, 50] and 0.8 to 8% of intracranial nerve sheath tumors [10, 38]. They develop from the sheaths of the trigeminal root, ganglion, or nerves. They usually appear at the level of Meckel’s cave, posterior fossa, or cavernous sinus and usually overlap multiple cranial fossae. Clinically, patients usually present with trigeminal nerve dysfunction, the most common symptom being trigeminal neuralgia (TN) [52]. Other common symptoms are numbness or burning sensation along the distribution of the nerve or one of its branches. Long-standing TS may also present with motor symptoms, such as masticatory disturbance and deviation of the jaw, but also symptoms of oculomotor nerves compression [38, 39].

Complete microsurgical resection remains challenging, due to their close relationship to vascular structures in the cavernous sinus, Meckel’s cave, and the skull base while extending from the middle towards the posterior fossa and vice versa [38]. Consequently, radical resection can be associated with further morbidity and mortality [39].

Stereotactic radiosurgery (SRS) is considered a valuable therapeutic alternative for treating benign intracranial tumors, due to its minimal invasiveness as well as to its safety profile and efficacy on vestibular [14, 24, 34, 44, 45] and non-vestibular schwannomas [7, 29]. Twenty years ago, a first report by Huang et al. [16] evaluated the role of SRS by Gamma Knife (GK) in trigeminal nerve schwannomas. Since then, many institutions reported their results in treating this uncommon pathology [12–14, 19, 32, 40, 42, 50]. Biological behavior of different benign Schwann cell tumors is often considered similar, thus making SRS a valuable treatment option.

Here, we performed a systematic review and meta-analysis of the published series on SRS for TS. We were particularly interested in evaluating tumor control, as well as symptom improvement for patients with secondary trigeminal neuralgia (TN) [5]. The available case series show a significant variability in both tumor control and clinical results. Understanding how SRS impacts the symptomatic course of secondary TN patients is critical, allowing better patient selection. Moreover, this could potentially suggest a standardized method for outcome reporting. In this paper, we provide the first systematic review and meta-analysis of outcomes following SRS for TS, researching improvement of pretherapeutic secondary TN or diplopia after SRS in TS; there is no comparison to other therapeutic approach.

## Material and methods

### Article selection and data extraction

**Fig. 1:**
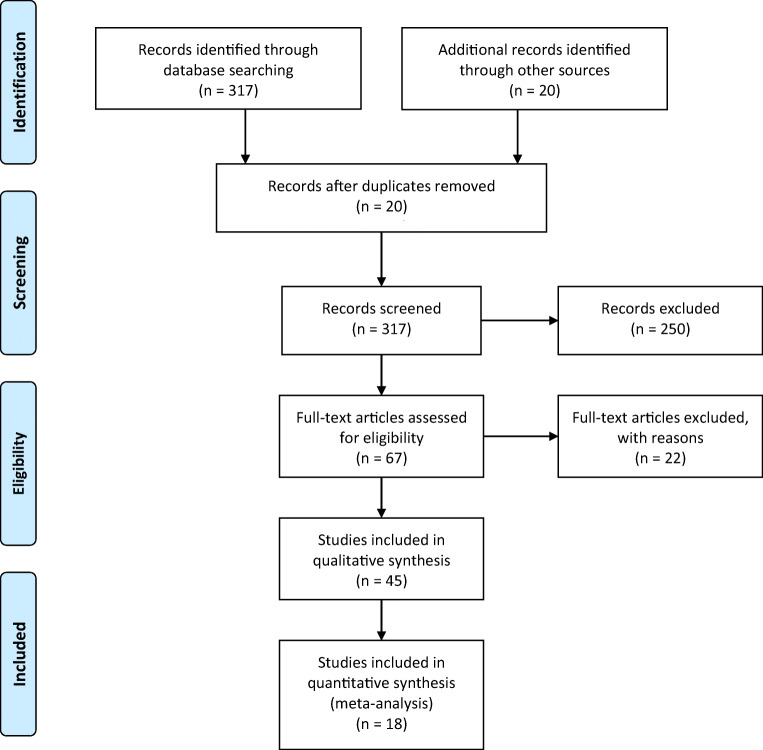
PRISMA flow-chart with study selection details

A PubMed search was performed for entries between January 1990 and December 2019 using the following query guidelines of January 1990 to December 2019: ((trigeminal AND (radiosurgery OR Gamma Knife)) AND (schwannoma)). We selected the 1990 as a starting date because prior to this date, there were few papers published in common indications on SRS, whereas TS was an uncommon one. Inclusion criteria required that each article be a peer-reviewed clinical study or case series of TS treated with SRS, independently of the device. As such, case reports, non-English studies, and conference papers or abstracts were excluded. Other exclusion criteria identified studies reporting non-vestibular schwannomas or TS treated with other radiation means than SRS (including radiotherapy). The article selection is illustrated in Fig. 1, which includes the studies reported subsequently in Tables 1 and 2. Two separate reviewers applied the inclusion criteria to the PubMed search result; there were no disagreements. Moreover, four separate reviewers applied the exclusion criteria to the remaining articles.

**Table 1:** Data related to tumor control

Series	No	Follow-up months (mean/median, range)	Anatomical location (Jefferson A, B, C)	Upfront SRS (*n*, %)	TV (cc) (mean/median, range)	Dose Gy (mean, range)	Tumor control (%)	Tumor decrease (%)
Kida et al. (1998) [19]	19	20.7 (12–20)	-	11 (57.9%)	-	14.7 (12–18)	100%	9/16 (56.3%) (16 reported)
Mabanta et al. (1999) [23]	7	-	-	7 (100%)	-	13.1	100%	-
Huang et al. (1999) [16]	16	44 (8–116)	9 (56.2%), 6 (37.5%), 1 (6.3%)	10 (62.5%)	5.3 cc (1–17.8)	15.3 (12–20)	100%	9/16 (56.3%)
Pollock et al. (2002) [32]	10	43* (12–111)	-	-	-	13	-	-
Pan et al. (2005) [28]	56	68 (27–114)	18 (34.6%), 9 (17.3%), 29 (51.8%)	42/56 (75%)	8.7 (0.8–33)	13.3 (10–15)	52/56 (93%)	48/56 (85.7%)
Sun et al. (2006) [43]	58	42.5 MRI	-	-	4.6	13.1	54/58 (93.1%)	38/58 (65.5%)
Hasegawa et al. (2007) [15]	37	54 (12–140)	17 (46%, 12 (32%), 8 (22%)	20/37 (54%)	10.3 (1.2–40.4)	14.2 (11–16)	32/37 (87%)	24/37 (65%)
Peker et al. (2007) [30]	15	61 (37–105)	-	11/15 (73.3%)	4 (0.9–17)	16 (14–22)	15/15 (100%)	13/15 (86.7%)
Phi et al. (2007) [31]	22	46 (24–89) clinic 37 (24–79) MRI	5/22 (22.7%), 5/22 (22.7%), 11/22 (50%) 1/22 middle and extracranial	18/22 (81.8%)	4.1 (0.2–12)	13.3 ± 1.3	21/22 (95%)	16/22 (73%)
Sheehan et al. (2007) [40]	25	48.5 (12–104)	-	18/25 (72%)	3.96 (0.63–8.5)	15 (10.2–17)	22/25 (88%)	12/25 (48%)
Kano et al. (2009) [18]	33	72 (7.2–147.9)	6/33 (18.2%), 17/33 (51.5%), 10/33 (30.3%)	22/33 (66.6%)	4.2 (0.5–18)	15 (12–20)	29/33 (87.9%)	17/33 (51.5%)
Champ et al. (2012) [6]	10	44.9 (19–120)	-	10/10 (100%)	4.8 (0.35–13.8)	13.3 (12–15)	9/10 (90%)	2/10 (20%)
Yianni et al. (2012) [50]	74	48.2 (6–168)	-	44/74 (59.4%)	5.3 (0.4–19.9)	16.4 (12–30)	69/74 (93.3%)	58/74 (78.4%)
Hasegawa et al. (2013) [12]	53	98 (4–241)	-	34/53 (64&)	6 (0.2–30)	14 (11–20)	46/53 (86%)	31/53 (58%)
Sun et al. (2013) [42]	52	61 (12–156)	14 (26.9%), 18 (34.6%), 18 (34.6%)	32/52 (61.5%)	7.2 (0.5–38.2)	13.9 (11–17)	45/52 (86.5%)	40/52 (75.9%)
Nettel et al. (2016) [27]	23	40 (12–146)		11/23 (47.8%)	4.5 (0.46–11.2)	14 (113–20)	20/22 (91%)	15/22 (68.2%)
Snyder et al. (2016) [41]	22	18.5 (3–143) clinical 27 (5–178) MRI	14 /22 (63.6%), -, 8/22 (36.4%)	19/22 (86.4%)	3.3 (0.2–10.7)	14.1 ± 1.4	17/22 (77.3%)	12/22 (54.6%)
Ryu et al. (2018) [37]	32	90.5 (49–281)		25/32 (78.1%)	5.3 (0.4–25.2)	13 (12–18)	27/32 (84.3%)	11/32 (34.3%)

**Table 2:** Data related to symptom improvement

Series	No	Symptom duration months (mean, range)	Symptom at discovery (facial pain, V^−th^ dysfunction)	Clinical improvement	Clinical stabilization	New or worsening deficits
Kida et al. (1998) [19]	19	-	18/19 (94.7%) facial dysesthesia 7/19 (36.8%) facial numbness 4/19 (21.1%) TN 2/19 (10.5%) diplopia	6/19 (31.6%) overall 3/4 (75%) TN	11/19 (57.9%)	2/19 (10.5%) worsened TN
Mabanta et al.(1999) [23]	7		7 (100%) facial pain 2/7 (28.6%) diplopia	-	-	-
Huang et al. (1999) [16]	16	26.4 (12–72)	15 (93.7%) facial hypesthesia 4/16 (25%) TN 3/16 (18.8%) diplopia	5/16 (31.3%) overall 3/15 (20%) hypesthesia 1/4 (25%) TN 1/3 (33.3%) diplopia	11/16 (68.7%)	0 (0%)
Pollock et al. (2002) [32]	10	-	-	-	-	3/10 (33.3%) trigeminal dysfunction 1/3 temporary masseter dysfunction
Pan et al. (2005) [28]	56	5 (0.5–12)	35/52 (63%) facial hypesthesia 12/52 (21%) diplopia (3 surgical) 9/52 (17.3%) TN 5/56 (9%) master weakness	14/56 (25%) overall 21/35 (60%) hypesthesia 4/9 (44.4%) TN 5/12 (41.7%) diplopia	-	5/35 (14.3%) facial numbness 1/56 (1.8%) masseter weakness 4/56 (7.1%) mild atrophy of masseter and temporal muscles
Sun et al. (2006) [43]	58		43/58 (74.1%) facial hypesthesia 13/58 (22.4%) TN 16/58 (27.6%) diplopia	28/58 (48.3%) overall 10/13 (76.9%) TN	23/58 (39.6%)	7/58 (12.1%)
Hasegawa et al. (2007) [15]	37	-	32/37 (86.5%) facial hypesthesia 9/37 (24.2%) TN 9/37 (86.5%) diplopia 14/37 (38%) master weakness	10/37 (27%) overall resolved 12/37 (40%) hypesthesia 6/9 (66.7%) TN 4/9 (44.4) diplopia	16/37 (46%)	5/37 (14%) tumor enlarged or uncontrollable facial pain appeared (surgery)
Peker et al. (2007) [30]	15	-	7/15 (46.7%) hypesthesia 3/15 (20%) TN	6/15 (40%) overall 2/3 (33.3%) TN 3/6 (50%) diplopia	8/15 (3.3%)	1/15 (6.7%) transient facial numbness and diplopia
Phi et al. (2007) [31]	22	-	11/22 (50%) facial hypesthesia 11/22 (50%) TN	4/11 (36.4%) hypesthesia 8/11 (72.7%) TN	8/22 (36.4%)	6/22 (27%)
Sheehan et al. (2007) [40]	25	-	16/44 (64%) facial hypesthesia 11/25 (44%) TN	18/25 (72%) overall 7/11 (64%) TN 9/11 (82%) diplopia	4/25 (16%)	3/25 (12%)
Kano et al. (2009) [18]	33	-	7/33 (21.2%) trigeminal neuropathy 8/33 (24.2%) TN	11/33 (33.3%) overall 7/16 (43.7%) hypesthesia 2/8 (25%) TN 5/9 (55.5%) diplopia	19/33 (57.6%)	3/33 (9.1%)
Champ et al. (2012) [6]	10	-	7/10 (70%) trigeminal neuropathy 3/10 (30%) TN	1/6 (16.7%) hypesthesia 1/3 (33.3%) TN 1/2 (50%) diplopia	6/10 (60%)	1/10 (10%)
Yianni et al. (2012) [50]	74	-	59/74 (79.7%) trigeminal neuropathy 10/74 (13.5%) TN 18/74 (24.3%) diplopia	5/10 (50%) TN 3/18 (16.7%) diplopia	-	5/74 (6.7%)
Hasegawa et al. (2013) [12]	53	-	43/53 (81%) trigeminal neuropathy 16/53 (30%) TN 10/53 (19%) diplopia	20/41 (49%) overall 18/39 (46%) facial hypesthesia 9/12 (75%) TN 3/9 (33%) diplopia	-	8/53 (15.1%)
Sun et al. (2013) [42]	52	-	29/52 (55.8%) facial hypesthesia 11/52 (21.2%) jaw weakness 10/52 (19.2%) TN 4/52 (7.7%) diplopia	35/52 (65.3%) overall 20/52 (69%) facial hypesthesia 9/10 (90%) TN 8/11 (72.7%) jaw weakness	14/52 (26.9%)	2/52 (3.8%)
Nettel et al. (2016) [27]	23	-	11/23 (47.8%) facial hypesthesia 6/23 (26.1%) diplopia 3/23 (13%) TN	12/23 (52%) overall	9/23 (39%)	2/23 (8.7%)
Snyder et al. (2016) [41]	22	-	15/22 (68.2%) facial paresthesia 15/22 (68.2%) trigeminal dysfunction 8/22 (36.4%) TN 5/22 (22.7%) diplopia	8/22 (42.1%) overall 5/15 (33.3%) facial hypesthesia 4/8 (50%) TN	8/22 (42.1%)	5/22 (22.7%)
Ryu et al. (2018) [37]	32		26/32 (81.3%) facial hypesthesia 11/32 (34.4%) TN 7/32 (21.9%) diplopia	12/26 (46.2%) facial hypesthesia 9/11 (81.8%) TN 2/7 (28.6%) diplopia		

This study was performed in accordance with the published Preferred Reporting Items for Systematic Reviews and Meta-Analyses (PRISMA) guidelines [26].

In extracting data from these studies, we paid attention to the diagnosis modality and clinical and neuroimaging classifications. In most series, neuroimaging diagnosis in patients without previous surgery was based upon classical characteristics of extra-axial uniformly enhancing tumors, involving the middle and/or posterior fossa, without any evidence of dural tail, accompanied by predominant clinical signs of trigeminal nerve dysfunction [35]. Clinical assessment was not reported using particular scales, especially in the context of TN. Jefferson’s classification scheme was used for neuroimaging extensions: A, predominantly middle fossa; B, posterior fossa; and C, dumbbell-shaped lesion involving both the middle and posterior fossa [17]; based upon the relationship with the brainstem, the classifications were types I (no compression of the brainstem), II (brainstem compression without deviation of fourth ventricle), and III (deviation of fourth ventricle).

We extracted data related to trigeminal dysfunction, TN, diplopia, tumor control, regression, stability, and progression before and after SRS.

### Statistical analysis using OpenMeta (Analyst) and random-effects model

Due to the high variation in study characteristics, a statistical analysis using a binary random-effects model (DerSimonian-Laird method) was performed. We used OpenMeta (Analyst) from the Agency for Healthcare Research and Quality.

Weighted summary rates were determined using meta-analytical models. Testing for heterogeneity was performed for each meta-analysis.

Pooled estimates using meta-analytical techniques were obtained for all the outcomes previously described in the same section.

## Results

### Study selection

This meta-analysis included 18 studies, comprising a total of 564 patients [6, 12, 15, 16, 18, 19, 23, 27, 28, 30–32, 40–43, 50]. Among those, only one reported the outcomes of linear accelerators (Linac) [23] while the others of GK.

### Study characteristics

The detailed study characteristics can be seen in Tables 1 and 2.

### Tumor control after SRS

**Fig. 2:**
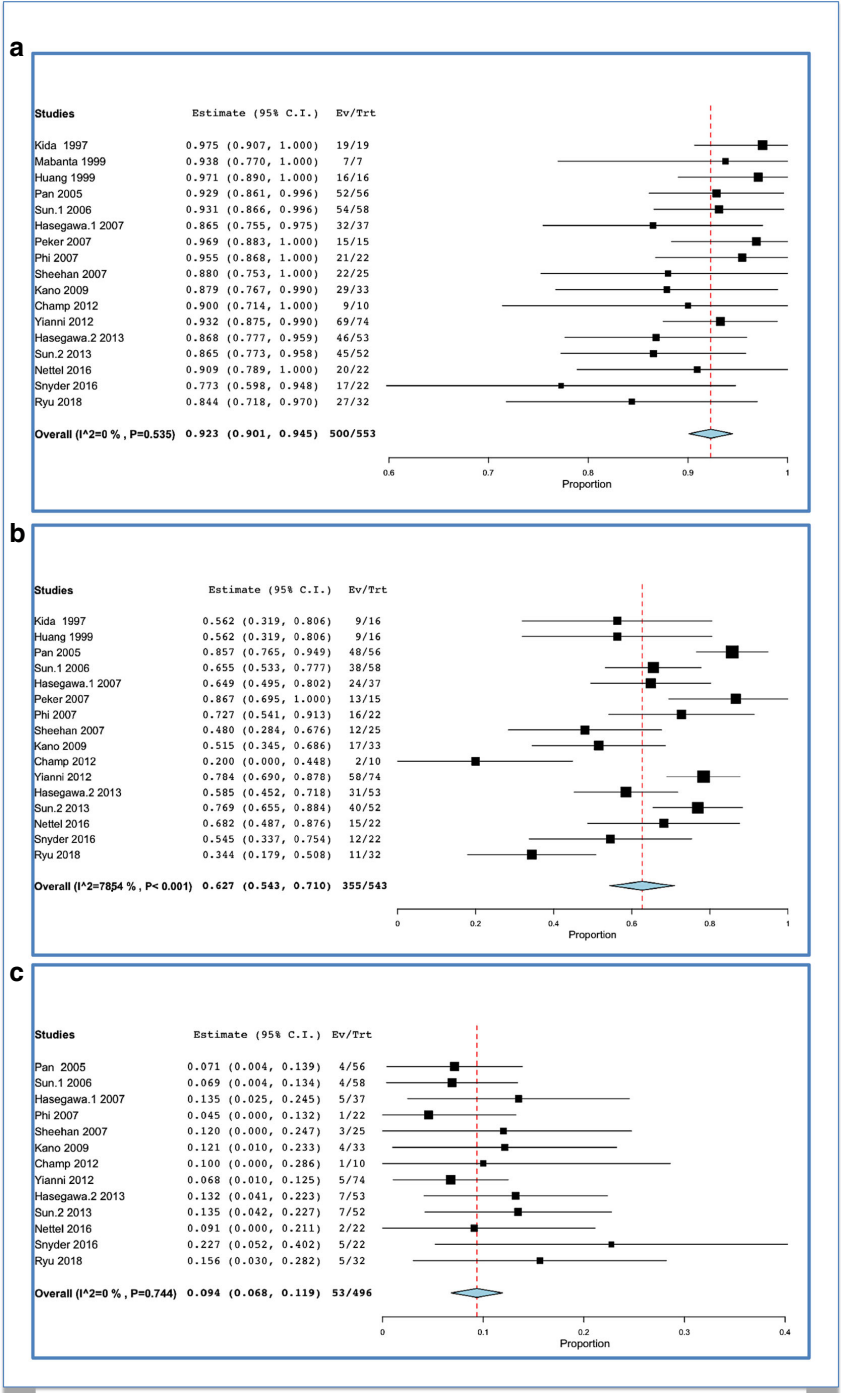
Tumor control rates after SRS for TS: **a** tumor control (including stability and decrease in volume); **b** tumor decrease; **c** tumor progression rates

Tumor stability or regression after SRS was described in 500 out of 553 of the reported patients, which corresponds to a rate of 92.3% (range 90.1–94.5; I^2 = 0; *p* heterogeneity = 0.53; *p* < 0.001; Fig. 2a). Factors involved in better tumor control were as follows: anatomical location (for Jefferson types A, B, and C were 93, 75, and 86% at 5 years; for I, II, and III were 100, 87, and 50%, respectively) [15]; root or ganglion tumor type [18]; female sex or < 8 cc tumor volume [18]; marginal and maximal dose [12]; and target volumes of less than 5 cc [37]. Tumor expansion was associated with higher prescribed doses (mean 14.9 versus 13.6 Gy) and with further lack of tumor control [41]. Transient tumor expansion was also associated with cystic components [37].

### Tumor decrease after SRS

Tumor decrease or regression was encountered in 355 out of the 543 reported patients, which corresponded to a rate of 62.7% (range 54.3–71; I^2 = 78.5; *p* heterogeneity and *p* < 0.001; Fig. 2b).

### Tumor progression after SRS

Tumor progression was encountered in 53 out of the 496 cases, which corresponded to a rate of 9.4% (range 6.8–11.9; I^2 = 0%; *p* heterogeneity = 0.74; *p* < 0.001; Fig. 2c).

### Overall clinical improvement

**Fig. 3:**
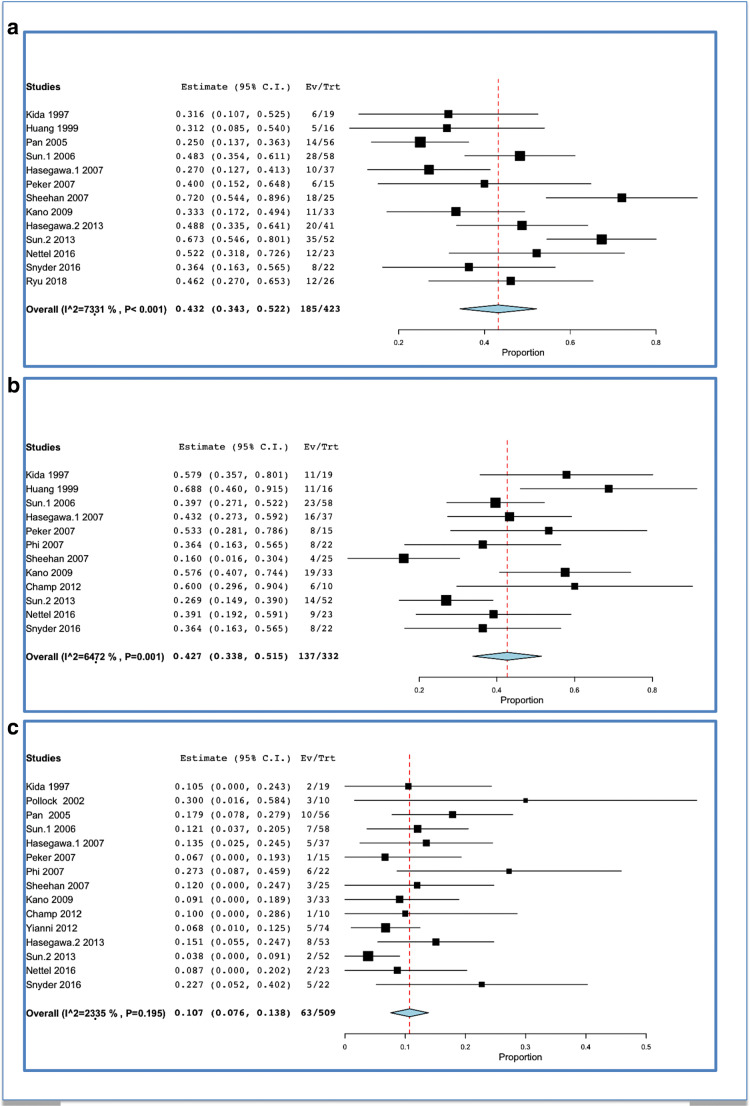
Clinical course after SRS for TS: **a** overall improvement; **b** stabilization; **c** worsening

Overall clinical improvement was encountered in 185 out of 423 patients, which corresponded to a rate of 43.2% (34.3–52.2; I^2 = 73.31%; *p* heterogeneity and *p* < 0.001; Fig. 3a).

### Overall clinical stabilization

Clinical stabilization was encountered in 137 out of 332 patients, which corresponded to a rate of 42.7% (33.8–51.5; I^2 = 64.72%; *p* heterogeneity = 0.001, *p* < 0.001; Fig. 3b).

### Overall clinical worsening

Clinical worsening (Fig. 3a) was reported in 63 out of 509 cases, which corresponded to a rate of 10.7% (7.6–13.8; I^2 = 23.35%; *p* heterogeneity = 0.195 and *p* < 0.001; Fig. 3c). Cranial neuropathies have been considered in some studies as associated with loss of central enhancement, tumor expansion, and tumor location extended in the cavernous sinus [31]. Symptom worsening was classically associated with transient tumor expansion [31].

### Clinical improvement of TN

**Fig. 4:**
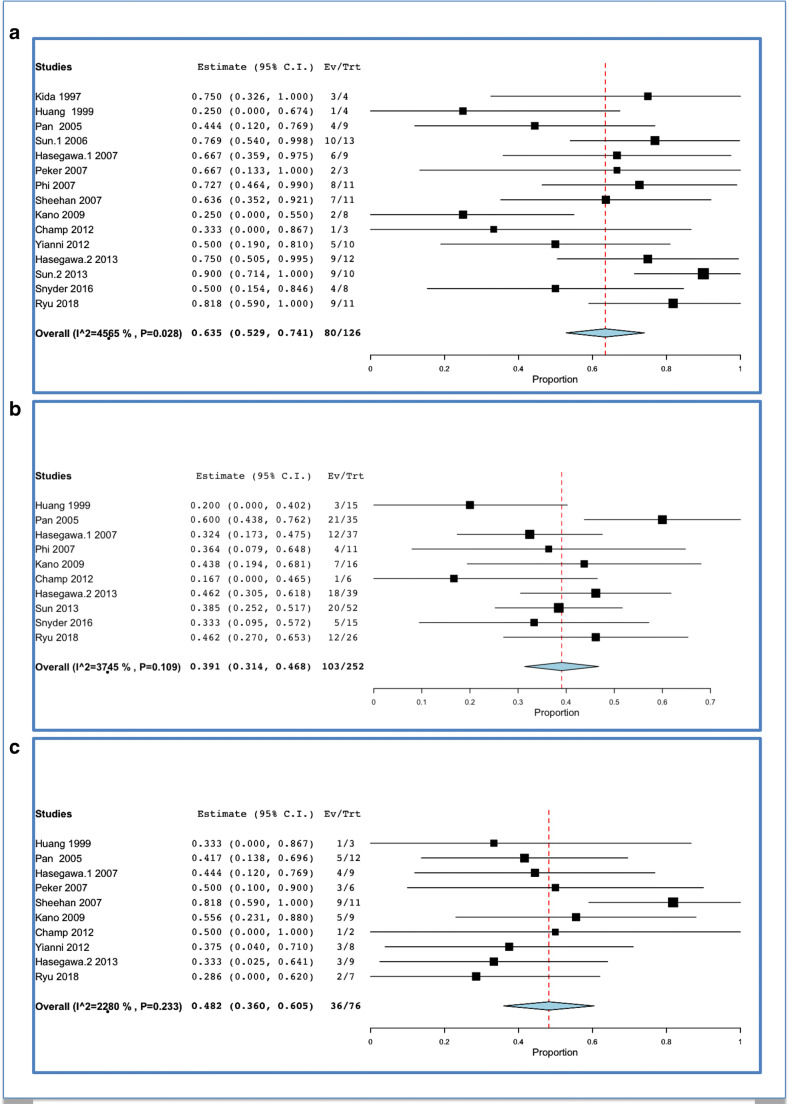
Specific outcomes after SRS: **a** trigeminal neuralgia; **b** trigeminal hypesthesia; **c** oculomotor

Clinical improvement of trigeminal neuralgia was encountered in 80 out of 126 patients, which corresponded to a rate of 63.5% (52.9–74.1; I^2 = 45.65%; *p* heterogeneity = 0.028, *p* < 0.001; Fig. 4a).

### Clinical improvement of facial hypoesthesia

Clinical improvement of facial hypoesthesia was reported in 103 out of 252 cases, which corresponded to a rate of 39.1% (31.4–46.8; I^2 = 37.45%; *p* heterogeneity = 0.109 and *p* < 0.001; Fig. 4b).

### Clinical improvement of diplopia

Clinical improvement of diplopia was reported in 36 out of 76 cases, which corresponded to a rate of 48.2% (36–60.5; I^2 = 22.8%; *p* heterogeneity = 0.233 and *p* < 0.001; Fig. 4c).

### Adverse reactions

Most commonly reported adverse reactions were pseudoprogression (variable as reported by series and based upon neuroimaging), ranging from 2.2% [30] to 37.5% [16]; cranial nerve V dysfunction (30%) [32]; expansion/enlarged cyst (11%) or increased pain (10%) [12]; cranial nerve dysfunction (8.6%) [27]; pseudocapsule formation [40]; and hydrocephalus (3.1%) [37] as reported individually in each of the series.

## Discussion

In this study, we provide a meta-analysis and systematic review of tumor control and symptomatic improvement after SRS for TS. This meta-analysis included 18 studies comprising a total of 564 patients. Tumor control rates after SRS were as high as 92.3% and tumor decrease rates 62.7%, while tumor progression rates 9.4%. Clinical improvement rates of trigeminal neuralgia were 63.5%, and oculomotor nerves improvement rates were 48.2%.

Historical standard treatment for TS was microsurgical resection. As many other benign skull-base pathologies, TS have been once considered forbidding tumors for microsurgical resection due to high rates of morbidity and mortality. Nowadays, radical resection is considered feasible, using a combination of skull-base approaches and microsurgery. However, neuropathies following surgery vary between 6.6 and 86% [1, 3, 25, 38]. The major challenges related to complete microsurgical resection are injury of cavernous sinus components (including carotid artery or the abducens nerve) and management of brainstem adherence of tumor capsule, in cases with posterior fossa extension [9, 38, 39]. The largest surgical series was published by Konovalov et al. [22], who reported 111 cases. The authors achieved radical removal in 86 patients (77.5%), with symptomatic recurrence in 13 (11.7%) cases. Fukushima described a series of 38 patients, with radical removal in 30 (78.9%) out of 38 cases, without any perioperative death [9]. Samii et al. reported the total removal in 10 (83.3%) of 12 patients [38]. Both cases with subtotal resection developed tumor progression. Postoperative complications included tetraparesis or facial nerve palsy. Al-Meftyet al. [1] reported a surgical series of 25 cases, all involving the cavernous sinus. Preoperative trigeminal nerve sensory deficit improved in 44% of cases and facial pain decreased in 73%. Three cases had tumor recurrence, with one experiencing another 2 surgeries [1]. Three patients developed cranial nerve neuropathies. Further skull-base approaches have been developed, providing better tumor exposure with minimal brain retraction without increased risk of morbidity. Recent study series by Goel et al. [11] reported a total resection rate in 73% of cases. Mortality rates have progressively declined from as high as 41% before 1956 to 25% before 1970 and further decreasing to 5.3% [3, 39] or even to 2.7% in more recent series [11]. Radical resection remains challenging, even in experienced hands, despite the most recent advances in skull-base surgery and neuromonitoring. Progression rates after subtotal resection, without adjuvant treatments, range from 12 to 35.7% [1, 3, 25, 38].

The current literature contains few series reporting the role of fractionated radiotherapy (FRT) in this pathology. Wallner et al. [47] reported 8 cases, with a tumor control rate of 50%. The administrated dose was 45–54 Gy, with 1.6 to 1.8 Gy/fraction. Another series by Zabel et al. [51] reported 13 patients, treated with 57.6 Gy, with 100% tumor control, with one mild worsening of preexisting trigemina hypesthesia.

A unique comparison between SRS and FRT [6] revealed higher toxicity in the FRT group (38.5 versus 0%), although lesions treated with FRT had higher volumes (mean 9.5 versus 4.8 cc).

Currently and during the past 20 years, SRS has been primarily used as a primary or second line treatment [16]. The mechanism of action of SRS in schwannomas has been considered a combination between direct tumoricidal effects and delayed intratumoral vascular obliteration, as reported by in vitro experiments [2]. For example, in vestibular schwannomas, the used radiation doses were initially higher, followed subsequently by dose escalation, with similar tumor control rates and better clinical outcomes [20]. Large tumors can be treated using subtotal or gross total resection followed by GKR, as previously published in non-vestibular [8, 29, 49] and vestibular schwannomas [8, 46]. Other approaches might include staged volume radiosurgery, in selected cases [7]. Challenging aspects might be related to the proximity with the optic apparatus. It was initially considered that a maximal delivered dose less than 8 Gy should be kept; however, recent trials suggested that this dose could be safely increased [33]. Cystic benign tumors have been initially considered less responsive to SRS. Nevertheless, this myth has been recently invalidated [4]. In case of tumor progression, further SRS versus microsurgical resection, depending on the volume, remain viable options. Hydrocephalus can be managed with ventriculo-peritoneal shunt. The question concerning trigeminal dysfunction after GKR for TS remains open, especially as the doses used here are much lower as compared to those used in idiopathic TN [21]. The risk of malignancy after SRS is currently considered low in all available long-term follow-up [48].

Lastly, one must take into account that SRS and particularly GKR have had an important technique refinement. In fact, some of the patients reported here have been treated with crude dosimetric algorithm, including KULA (Elekta Instruments, AB, Sweden). In this respect, the clinical and radiological results have significantly improved and they will keep on improving.

### Limitations

There are several limitations to consider in our investigation. The first one is related to the innate shortcomings of meta-analysis techniques in neurosurgical studies. The second one refers to the fact that data were aggregated from multiple trials to generate a larger patient study group. While this type of approach enhances detection of statistically significant correlations between different parameters, the validity of the final data depends on the data quality collected by other authors and might be susceptible to selection bias. The third limitation is the number of patients which has been reported for some of the outcomes, with several studies lacking to report all data. The fourth limitation is the lack of scale reporting, in particular for trigeminal neuralgia, such as the Barrow Neurological Institute scale [36].

## Conclusion

Stereotactic radiosurgery is safe and effective for TS. Tumor control rates are as high as 92.3% and tumor decrease rates are 62.7%, while tumor progression rates are 9.4%. Clinical improvement rates of trigeminal neuralgia are 63.5%, and oculomotor nerves improvement rates are 48.2%. Future studies should report complete clinical evaluations, before and after SRS, using standardized scales.
